# Discovery of a colossal slickhead (Alepocephaliformes: Alepocephalidae): an active-swimming top predator in the deep waters of Suruga Bay, Japan

**DOI:** 10.1038/s41598-020-80203-6

**Published:** 2021-01-25

**Authors:** Yoshihiro Fujiwara, Masaru Kawato, Jan Yde Poulsen, Hitoshi Ida, Yoshito Chikaraishi, Naohiko Ohkouchi, Kazumasa Oguri, Shinpei Gotoh, Genki Ozawa, Sho Tanaka, Masaki Miya, Tetsuya Sado, Katsunori Kimoto, Takashi Toyofuku, Shinji Tsuchida

**Affiliations:** 1 Research Institute for Global Change, Japan Agency for Marine-Earth Science and Technology (JAMSTEC), Yokosuka, Kanagawa Japan; 2 Fish Section, Australian Museum, Sydney, NSW Australia; 3 School of Marine BioSciences, Kitasato University, Sagamihara, Kanagawa Japan; 4 Institute of Low Temperature Science, Hokkaido University, Sapporo, Hokkaido Japan; 5 Research Institute for Marine Resources Utilization, Japan Agency for Marine-Earth Science and Technology (JAMSTEC), Yokosuka, Kanagawa Japan; 6 Department of Marine Electronics and Mechanical Engineering, Tokyo University of Marine Science and Technology, Koto-ku, Tokyo, Japan; 7 TechnoSuruga Laboratory Co., Ltd., Shizuoka, Shizuoka Japan; 8 Department of Marine Biology, Tokai University, Shizuoka, Shizuoka Japan; 9 Department of Ecology and Environmental Sciences, Natural History Museum and Institute, Chiba, Chuo-ku, Chiba, Japan; 10 Institute for Extra-cutting-edge Science and Technology Avant-garde Research (X-star), Japan Agency for Marine-Earth Science and Technology (JAMSTEC), Yokosuka, Kanagawa Japan

**Keywords:** Biodiversity, Stable isotope analysis, Phylogenetics, Taxonomy, Ichthyology

## Abstract

A novel species of the family Alepocephalidae (slickheads), *Narcetes shonanmaruae*, is described based on four specimens collected at depths greater than 2171 m in Suruga Bay, Japan. Compared to other alepocephalids, this species is colossal (reaching ca. 140 cm in total length and 25 kg in body weight) and possesses a unique combination of morphological characters comprising anal fin entirely behind the dorsal fin, multiserial teeth on jaws, more scale rows than congeners, precaudal vertebrae less than 30, seven branchiostegal rays, two epurals, and head smaller than those of relatives. Mitogenomic analyses also support the novelty of this large deep-sea slickhead. Although most slickheads are benthopelagic or mesopelagic feeders of gelatinous zooplankton, behavioural observations and dietary analyses indicate that the new species is piscivorous. In addition, a stable nitrogen isotope analysis of specific amino acids showed that *N. shonanmaruae* occupies one of the highest trophic positions reported from marine environments to date. Video footage recorded using a baited camera deployed at a depth of 2572 m in Suruga Bay revealed the active swimming behaviour of this slickhead. The scavenging ability and broad gape of *N. shonanmaruae* might be correlated with its colossal body size and relatively high trophic position.

## Introduction

Marine environments are increasingly being affected by global climate change and anthropogenic activities, leading to oceanic warming, acidification, and deoxygenation even in deep-sea regions^1^. Such global changes are assumed to initially affect large, predatory consumers and subsequently have repercussions for organisms at lower trophic levels^2^. There is thus an urgent need to elucidate the present biodiversity and abundance of predatory fishes inhabiting deep-sea sites^3^.

In 2016, we conducted bottom longline surveys and in situ camera observations in Suruga Bay, Japan, with the aim of obtaining information on the faunal diversity and trophic interactions of deep-sea predatory fishes. Suruga Bay, which is the deepest bay in Japan (maximum depth: ca. 2500 m), is located in the central region of the Pacific coastline of the Japanese Archipelago. Since the late 1800s, natural history studies have been conducted in this bay and the fauna inhabiting the bay have accordingly been well documented^4–9^. Commercial deep-sea trawl, bottom longline, and baited pot fishing have been actively conducted in this bay and tons of predatory fish are caught annually. As such, in terms of gaining an understanding of the vulnerability of deep-sea ecosystems to anthropogenic impacts, Suruga Bay represents an ideal research location for constructing dynamic ecosystem models that incorporate large predators.

The genus *Narcetes* Alcock, 1890, is a member of the family Alepocephalidae (slickheads), consisting of three named species, i.e., *N. erimelas*, *N. lloydi*, and *N. stomias*^10^. The members of this genus are bathypelagic, inhabiting depths between 700 and 2600 m, and are distributed globally. They reach approximately 75 cm in standard length. This genus is characterized by multiserial teeth on premaxillary and dentary, laterally expanded dentary with a toothless area at symphysis. The members have 26–33 precaudal vertebrae and 18–25 caudal vertebrae (48–56 in total), with 3–14 more precaudal than caudal vertebrae, relatively large centra spaces for the spinal cord, 8–14 long pyloric caeca, generally 8 branchiostegal rays, and 3–6 + 1 + 10–17 gill rakers on the first arch^11^. Sazonov^10^ reviewed all species of the genus and synonymized *Bathytroctes alveatus* Garman, 1899 with *N. erimelas* Alcock, 1890 and *N. kamoharai* Okamura, 1984 and *N.* *wonderi* Herre, 1935 with *Narcetes lloydi* Fowler, 1934.

Surprisingly, during our surveys, we captured four individuals belonging to an extraordinarily large, unidentified *Narcetes* species from a region deeper than 2171 m at the opening of the bay. Although fishes in the order Alepocephaliformes are widely distributed in the world’s oceans and comprise a diverse group of deep-sea fishes^11–13^, their biology and systematics remain poorly understood, owing largely to the infrequent surveying of their deep habitats.

Here, we describe a novel slickhead species of the genus *Narcetes* and present detailed morphological descriptions, along with the results of X-ray computed tomography (CT), molecular phylogeny, and stable isotope analyses. This species described here is the largest alepocephalid fish reported to date and is one of the largest bathyal species of bony fish ever described, reaching ca. 140 cm in total length and ca. 25 kg in weight. The noteworthy trophic position, diet, and swimming behaviour of the newly discovered species are also reported.

## Results

### Bottom longline sampling

**Figure 1:**
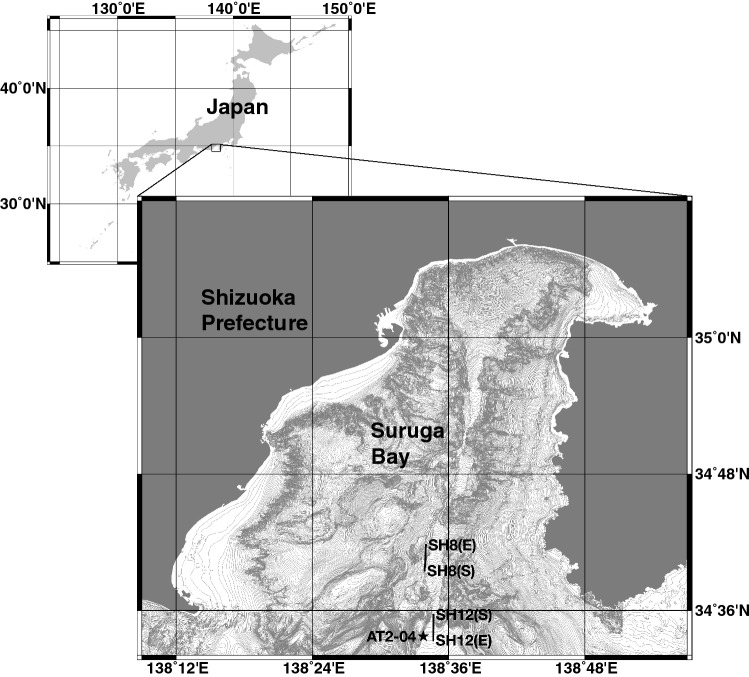
Research area and location of each longline and baited camera deployment. This map was created using QGIS software version 2.14.10 (https://qgis.org/) and bathymetric data M7001 supplied by the Japan Hydrographic Association (https://www.jha.or.jp/en/jha/). Solid line: longline tracks of SH8 and SH12; S and E represent the start and end locations of longlines, respectively. Solid star: deployment location of baited camera (AT2-04). This map is under copyright © 2018 Yoshihiro Fujiwara.

Four individuals of an alepocephalid species were collected from Suruga Bay using a bottom longline installed on the training vessel *Shonan maru* (SH) belonging to the Kanagawa Prefectural Marine Science High School. The first two individuals were collected at depths of 2171 m and 2179 m on 4th February 2016 during the cruise SH16-01, and the other two specimens were captured at depths of 2572 m and 2551 m on 23rd November 2016 during the cruise SH16-02 (Fig. 1, Table 1). The total lengths of the fish ranged from 122 to 138 cm (Table 1). The two individuals collected during the SH16-01 cruise were fixed with formalin as the holotype (sample ID: SH8-69) and paratype 1 (sample ID: SH8-43). The remaining two individuals (sample IDs: SH12-1 and SH12-14) collected during the SH16-02 cruise were frozen and were also deposited as paratypes 2 and 3, respectively (Table 1).

**Table 1:** Sampling dates and depths for the captured *Narcetes shonanmaruae* specimens.

Sample ID	SH8-69	SH8-43	SH12-1	SH12-14
Sampling date	4th Feb. 2016	4th Feb. 2016	23rd Nov. 2016	23rd Nov. 2016
Longline no.	SH8	SH8	SH12	SH12
Depth (m)	2171	2179	2572	2551
Total length (cm)	138.0	122.0	125.0	132.0
Weight (kg)	22.6	14.8	19.0	24.9
Status	Holotype	Paratype 1	Paratype 2	Paratype 3
Catalogue number/deposited institution	1160053133/Japan Agency for Marine-Earth Science and Technology	KPM-NI 43548/Kanagawa Prefectural Museum of Natural History	NSMT-P 132616/National Museum of Nature and Science	CBM-ZF-0019048/Natural History Museum and Institute, Chiba

Depth information was derived from the data logger records closest to each hooked fish.

### Systematics

Family Alepocephalidae Bonaparte, 1846.

Genus *Narcetes* Alcock, 1890.

*Narcetes shonanmaruae* sp. nov. Poulsen, Ida, Kawato & Fujiwara (Figs. 2 and 3, Tables 1 and 2, Supplementary Figs. S1–S16) (proposed English name: Yokozuna Slickhead, proposed Japanese name: Yokozuna Iwashi).

**Figure 2:**
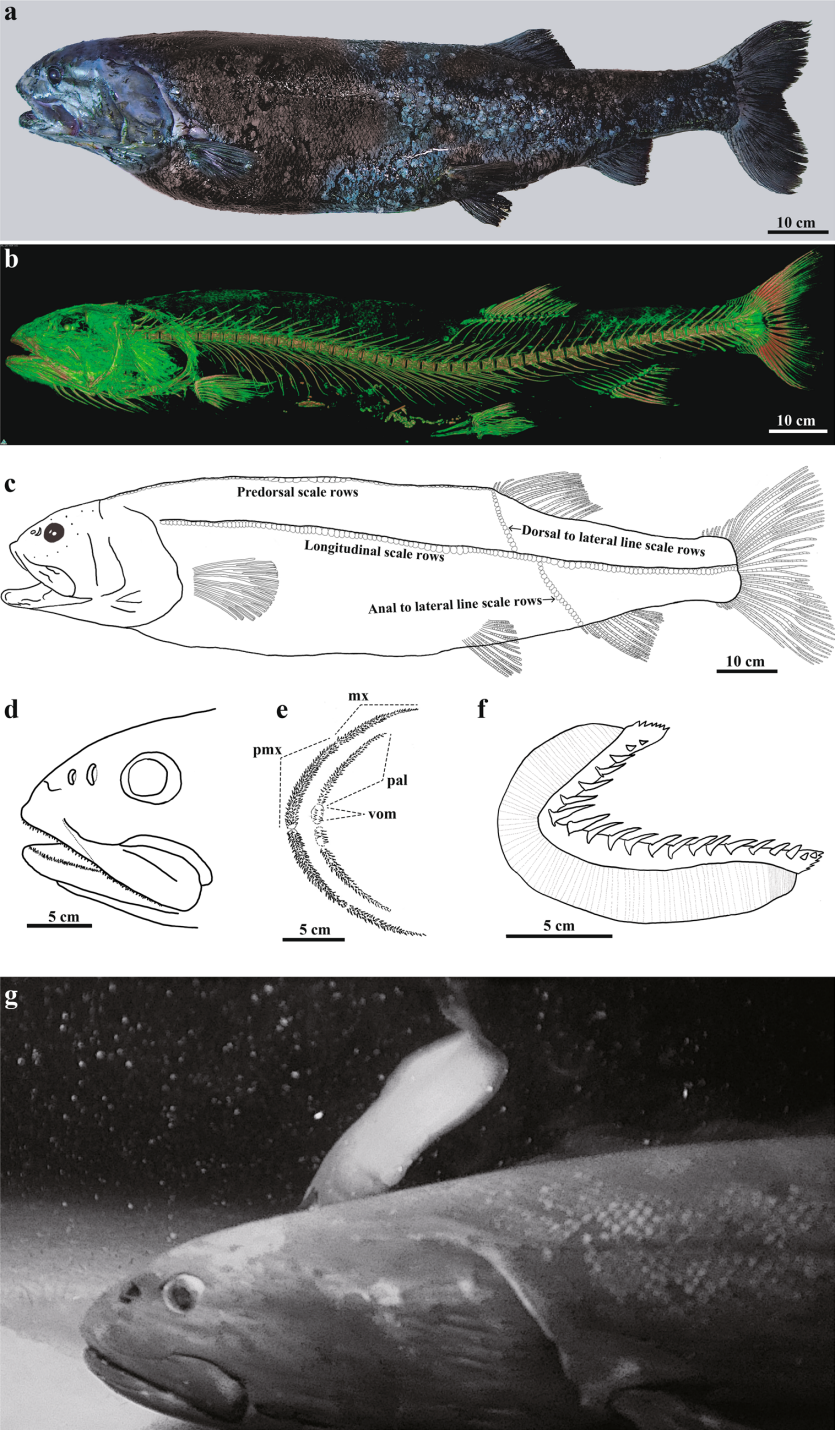
*Narcetes shonanmaruae*. (**a**), (**b**), (**d**), (**e**) holotype (SH8-69). (**c**) paratype 3 (SH12-14). (**f**) paratype 2 (SH12-1). (**a**) Lateral view. (**b**) Computed tomography image of the endoskeleton. (**c**) Lateral view. Scale rows indicated. Lateral line scales not shown. (**d**) Lateral view of head. (**e**) Tooth pattern of upper jaw. mx: maxillary, pal: platine, pmx:premaxillary, vom: vomer. (**f**) Gill raker. Right side of first arch. (**g**) In situ video grab of active swimming individual recorded using a baited camera.

**Figure 3:**
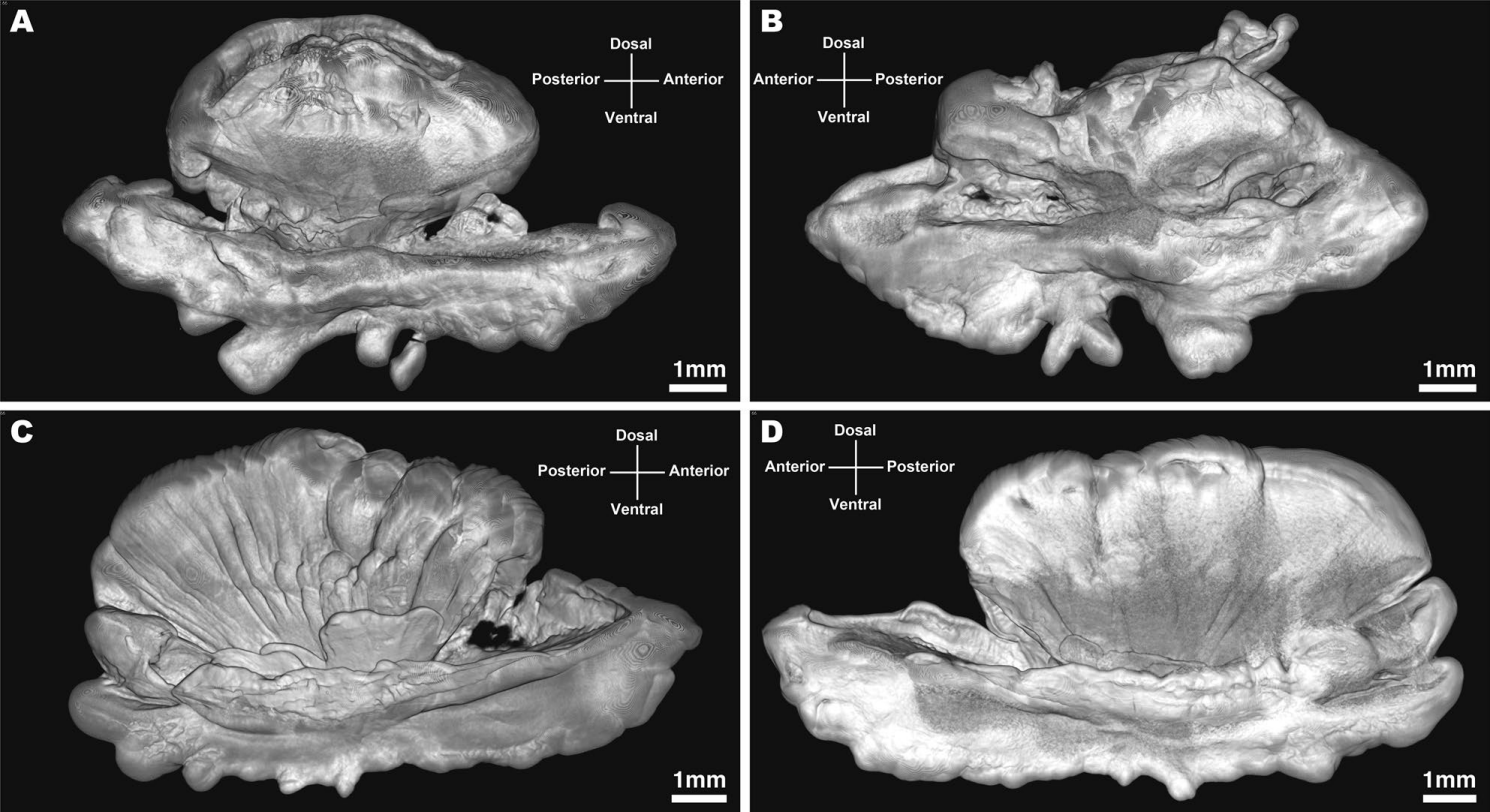
*Narcetes shonanmaruae*. Micro-computed tomography images of otoliths. (**a**) and (**b**) Holotype (SH8-69). (**c**) and (**d**) Paratype 1 (SH8-43). (**a**) and (**c**) Medial view of left otolith. (**b**) and (**d**) Medial view of right otolith. Scale bar: 1 mm.

**Table 2:** Morphometric and meristic information on *Narcetes shonanmaruae* and relatives*.*

	*Narcetes shonanmaruae*					*Narcetes erimelas*			*Narcetes lloydi*	*Narcetes stomias*
	SH8-69	SH8-43	SH12-1	SH12-14	Species range	NSMT-P63883	*Bathytroctes alveatus* syntype	Species range	Species range	Species range
Standard length (SL) (cm)	121.0	104.0	111.5	117.5	104.0–121.0	21.8	~ 16.1	13.7–74.7	9.2–50.0	23.8–57.5
**In % SL**										
Predorsal length	61.2	60.6	61.4	62.1	60.6–62.1	64.7	–	61.8–65.7	58.9–66.7	61.5–66.4
Preanal length	76.3	73.4	77.9	78.3	73.4–78.3	80.3	–	76.4–81.4	67.9–78.1	67.6–74.8
Prepelvic length	59.9	58.5	60.3	64.9	58.5–64.9	61.5	(55.6)	61.2–63.2	49.9–58.8	50.5–58.8
Ventroanal length	16.8	15.8	20.4	18.5	15.8–20.4	15.6	–	14.4–18.3	–	–
Dorsal fin base length	12.2	12.2	12.1	12.6	12.1–12.6	14.3	–	13.1–16.9	13.4–19.4	13.4–17.6
Anal fin base length	7.6	7.3	7.7	8.1	7.3–8.1	9.4	–	8–10.8	10.0–13.8	9.2–12.8
Maximum body depth	13.5	18.3	22.9	23.8	13.5–23.8	17.9	(16.7)	15.2–23.8	15.7–22.7	14.3–22.1
Caudal peduncle depth	7.6	8.0	7.8	7.5	7.5–8.0	7.7	–	6.4–8.4	5.8–9.4	6.0–9.7
Caudal peduncle length	12.1	10.3	12.1	11.1	10.3–12.1	10.1	–	10.1–15.4	15.7–23.9	17.1–22.4
Postorbibal width of head	9.8	9.0	8.9	8.6	8.6–9.8	9.5	–	9.5–12.5	8.6–13.4	8.9–11.3
Head length	24.4	27.4	24.7	24.5	24.4–27.4	36.3	34.2 (33.3)	28.9–38.8	26.6–36.6	26.8–32.1
Snout length	7.1	8.6	7.7	7.5	7.1–8.6	9.4	11.8 (10.0)	8.9–11.6	8.0–12.3	7.0–10.0
Orbit diameter	3.1	3.5	2.9	3.2	2.9–3.5	6.6	–	5.2–8.9	3.2–7.1	4.3–6.3
Interorbital distance	6.9	7.0	7.9	7.5	6.9–7.9	7.0	6.5 (6.7)	6.4–7.4	5.3–9.2	4.1–7.4
Upper jaw length	12.7	13.5	12.6	13.1	12.6–13.5	20.0	(> 16.7)	15.8–20.7	14.2–20.6	15.5–18.0
Lower jaw length	13.6	13.1	14.8	13.3	13.1–14.8	21.1	–	15.6–22.3	15.1–23.0	15.8–19.8
Dorsal-fin rays	14	14	14	14	14	15	15–16	15–17	17–22	17–21
Anal-fin rays	11	12	11	11	11–12	11	11	11–13	12?–18	14–18
Pectoral-fin rays	11	11	12	13	11–13	10	11	9–11	7–11	8–12
Pelvic fin rays	9	8	9	9	8–9	10	10(?)	8–10	8–10	7–10
Branchiostegal rays	7	7	7	7	7	??	–	7	8	8–9
Gill rakers on the first gill arch	6 + 16	5 + 14	6 + 15	6 + 17	[5, 6] + [14–17]	5 + 16^a^	5 + 16	[5, 6] + [15, 16]	[5, 6] + [15–18]	[3–5] + [11–15]
Predorsal scale rows	77	88	79	73	73–88	37	–	37–40	27–37	42–60
Longitudinal scale rows	118	125	118	116	116–125	65^b^	70	68–85	60–75	87–120
Dorsal to lateral line scale rows	18	19	19	18	18–19	10	7	7–12	8–11	9–12
Anal to lateral line scale rows	19	21	19	19	19–21	10	14?	10–14	9–12	9–14
Lateral line scale	60	54	58	62	54–62	NA	–	60	53–65	52–81
Pyloryc caeca	9	13	11	13	9–13	–	–	10–14	8–16	6–12
Precaudal vertebrae (incl. skull-connecting vertebrae)	28	28	–	–	28	30	32^c^	31–34	29–33	25–29
Caudal vertebrae (incl. vertebra articulated with parahypural)	21	21	–	–	21	22	19^c^	19–23	23–25	20–23
**Otolith (mm)**										
Anteroposterior length										
Left	10.9	12.3	–	–	–	–	–	–	–	–
Right	11.5	13.0	–	–	–	–	–	–	–	–
**Dorsoventral length**										
Left	6.3	6.8	–	–	–	–	–	–	–	–
Right	7.1	6.8	–	–	–	–	–	–	–	–
**Mediolateral length**										
Left	3.0	2.7	–	–	–	–	–	–	–	–
Right	3.2	2.6	–	–	–	–	–	–	–	–
References	Present study	Present study	Present study	Present study	Present study	Present study	Sazonov^10^, Present study	Garman^89^, Sazonov^10^, Present study	Sazonov^10^	Sazonov^10^

–: Data not shown due to lack of measurements.

^a^Right gill rakers counted due to malformation of left rakers.

^b^Right scale rows counted due to sample condition.

^c^Counted from syntype ID: MCZ 28477.

#### Material

Holotype: JAMSTEC No. 1160053133 (Sample ID: SH8-69), 121.0 cm SL, female, Suruga Bay, 2171 m depth, 4th February 2016, collected by YF, GenBank No. AP018429.

Paratypes: (1) KPM-NI 43548 (Sample ID: SH8-43), 104.0 cm SL, female, Suruga Bay, 2179 m depth, 4th February 2016, collected by YF, GenBank No. AP018430; (2) NSMT-P 132616 (Sample ID: SH12-1), 111.5 cm SL, female, Suruga Bay, 2572 m depth, 23rd November 2016, collected by YF, DDBJ No. LC409533; (3) CBM-ZF-0019048 (Sample ID: SH12-14), 117.5 cm SL, female, Suruga Bay, 2551 m depth, 23rd November 2016, collected by YF, DDBJ No. LC409534. The holotype and three paratypes provided genetypes according to Chakrabarty et al.^14^. This published study and the nomenclatural acts it contains have been registered in ZooBank, the online registration system for the ICZN. The ZooBank LSIDs (Life Science Identifiers) can be resolved and the associated information viewed through any standard web browser by appending the LSID to the prefix “http://zoobank.org/”. The LSID for this publication is: urn:lsid:zoobank.org:act:7C56487C-7E3F-4096-ACED-171DCD90C4D0.

#### Species diagnosis

Anal fin entirely behind the dorsal fin. Teeth on premaxillary, maxillary, and dentary multiserial. Predorsal scale rows greater than 70. Longitudinal scale rows greater than 110. Dorsal to lateral line scale row greater than 15. Anal to lateral line scale rows greater than 17. Branchiostegal rays 7. Dorsal-fin rays less than 15. Precaudal vertebrae less than 30. Epurals 2. Lengths of head, snout, upper and lower jaws, orbit diameter, dorsal and anal fin bases relatively short against standard length (SL).

#### Description (Figs. 2, 3, Tables 1, 2, Supplementary Figs. S1–S16)

General appearance (Figs. 2a–c, Supplementary Figs. S1, S2, S16): Body relatively long, fusiform, more or less laterally compressed, head robust and broad, body colour purplish, black pores scattered on head; skull and head region without scales; dorsal-fin base anterior to origin of anal fin base non-overlapping in vertical direction; dorsal-fin base slightly posterior to pelvic fin base; pectoral fin base situated ventrolaterally on body, slightly closer to ventral margin than to lateral line; adipose fin absent; thick adipose layer between skin and muscle (“slippery” or “fatty tissue” layer).

Fins (Fig. 2b, Supplementary Figs. S4–S10): dorsal-fin rays, 14 in holotype and all the paratypes (including anterior three small rays); anterior four rays unbranched, 4th longest with 12 pterygiophores in holotype and 14 pterygiophores in paratype 1; anal-fin rays, 11 in holotype and paratype 2 and 3 (including anterior two small rays), 12 in paratype 1; anterior 3 rays unbranched, 4th longest with 11 pterygiophores in holotype and paratype 1; pectoral-fin rays, 11 in holotype and paratype 1, 12 in paratype 2, 13 in paratype 3; pelvic-fin rays, 9 plus a splint bone in holotype, 8 plus a splint bone in paratype 1, and 9 in paratypes 2 and 3 (splint bone unconfirmed due to no CT image); principal caudal-fin rays, 10 + 9 in holotype and paratype 1, and 10 upper and 9 lower procurrent rays in holotype and 11 upper and 9 lower in paratype 1.

Osteology (Figs. 1a,b, 2b,d,e, Supplementary Figs. S1–S4, S11, S16f): precaudal vertebrae, 28 and caudal vertebrae, 21 in holotype and paratype 1, amphicoelous (hour-glass shaped); neural and haemal spines prominent, all associated vertebral bones fully ossified; supraneurals present, situated above 10 most anterior precaudal vertebrae, seven supraneurals in holotype, five in paratype 1; epipleurals begin on 2nd vertebral centra; two epurals in holotype and paratype 1; posterior ural centra turned upwards; post-temporal, supracleithrum, and cleithrum forming almost right angles, thereby ‘squaring’ the head; presence of mesocoracoid and post-cleithrum indeterminate; circumorbital bones absent; opercle relatively small, slightly rounded, convex, except in front of pectoral base (concave); premaxillary broad with minute teeth, elongated, reaching halfway along the maxillary; maxillary almost double length of premaxillary, even broader, with minute teeth; two supramaxillae; dentary broad, covered with minute teeth in several rows.

Teeth (Fig. 2d,e, Supplementary Fig. S16f): many sharp teeth present on dentary, premaxillary, maxillary, vomer, and palatines, appear unordered, multiserial rows (teeth sockets present); number of teeth subject to allometric growth; tooth patches present on 5th ceratobranchial bone.

Scales (Figs. 1a, 2a,c, Supplementary Fig. S16a,b, Table 2): body scales relatively small, deciduous, cycloid and numerous, longitudinal scale rows 116–125, predorsal scale rows 73–88; isthmus sharply demarcated by absence of scales anteriorly, abruptly scaled posteriorly; skin on cleithrum without scales, demarcating head and angle between cleithrum and pectoral base; scales absent on nape; lateral line scales elongated, drawn into tube, expanded posteriorly, especially towards the tail region; skull and head region visibly naked, also sharply demarcated by purplish colour.

Others (Fig. 2a–d,f, Table 2, Supplementary Figs. S11, S16c–f): Lateral line straight, running along entire body; caudal fin slightly emarginate (Fig. 2a); eyes round, small relative to body size 2.9–3.5% SL; aphakic gap absent; paired nostrils, nasal cavities large, rosettes present immediately anterior to eyes, posterior largest, anterior round and less than half size of posterior; snout short (7.1–8.6% SL); seven branchiostegal rays; gill rakers (19–23) large, widely spaced, without teeth; small, slender pseudobranch filaments (12–14) present; photophores and swim bladder absent; pyloric caeca (9–13) extremely large; ovary developed in paratypes 2 and 3; ground body colour pigmentation purplish with scale pockets dark, appearing blackish/purplish (typical slickhead colouration); head, mouth, and gill cavities purplish/bluish.

Otoliths (Fig. 3, Supplementary Figs. S12–15): Otolith morphology ‘sailboat’ type^15^, variable within and between individuals, elongate anteroposteriorly, robust, outer face convex; sulcus deeply incised, not divided into ostium and cauda; crista superior absent, crista inferior present; dorsal part fan-shaped, including radial notches, dorsal rim variable, large hollow only present in holotype; ventral part ‘boat shaped’, including several lobes on ventral rim; rostrum present, gradually rising towards anterior tip; antirostrum present (rounded); posterior end relatively pointed.

#### Remarks

*Narcetes shonanmaruae* sp. nov. is similar to *N. erimelas* in anal fin position being entirely behind the dorsal fin. However, our species differs from all the other *Narcetes* species based on the following morphological characters: length of dorsal fin base less than 13% SL; upper jaw length less than 14% SL; lower jaw length less than 15% SL; number of dorsal-fin rays less than 15; number of predorsal scale rows greater than 70; number of dorsal to lateral line scale rows greater than 16; number of anal to lateral line scale rows greater than 17.

#### Etymology

The species epithet *shonanmaruae* is a feminine noun in Latin, referring to the ship ‘*Shonan maru*’ from which the type materials were caught, honouring the vessel’s considerable contribution to deep-sea fish research in the area. The proposed Japanese vernacular name is ‘Yokozuna Iwashi’. This species belongs to the family Alepocephalidae, which is referred to as ‘Sekitori Iwashi’ in Japanese: ‘Sekitori’ meaning a sumo wrestler and ‘Iwashi’ meaning a sardine, thereby implying a massive sardine. The term ‘Yokozuna’ refers to the highest rank in sumo wrestling in Japan. Accordingly, we propose the name ‘Yokozuna Iwashi’ as being indicative of the large body size and the high trophic position of the newly described species. English vernacular name: Yokozuna Slickhead.

#### Distribution

Currently only known from Suruga Bay at depths deeper than 2100 m.

### Phylogenetic placement of *Narcetes shonanmaruae*

Mitogenomic DNA sequences for the *N. shonanmaruae* holotype (SH8-69) and paratype 1 (SH8-43), in addition to those for three species of *Conocara* and *Talismania antillarum*, were determined in the present study and deposited in nucleotide sequence databases under the accession numbers shown in Supplementary Table S1. Each translated amino acid sequence was aligned with homologues from 73 other teleost species (75 operational taxonomic units, OTUs, in total, accession numbers shown in Supplementary Table S1). The evolutionary models selected for the maximum likelihood (ML) analysis of each amino acid sequence of 13 mitochondrial protein-coding genes are shown in Supplementary Table S2. The aligned positions of the 13 proteins from each species combined had a total length of 3790 amino acids.

**Figure 4:**
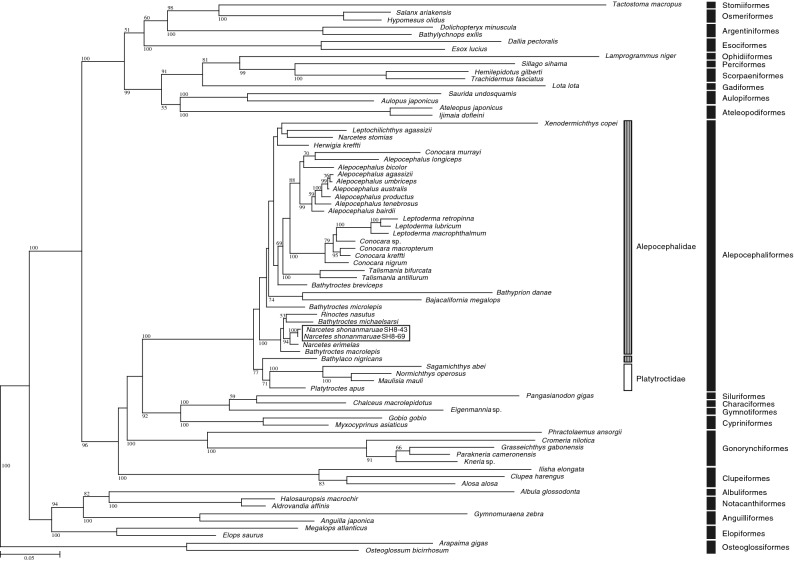
Phylogenetic placement of *Narcetes shonanmaruae* based on amino acid sequences from 13 protein-coding genes of the mitogenome. A maximum likelihood (ML) tree is shown. Scale bar represents 0.05 nucleotide substitutions per sequence position. Bootstrap values are shown for each branch. *Narcetes shonanmaruae* is highlighted.

The phylogenetic position of *N. shonanmaruae* determined from our ML analysis was distinct from that of all other alepocephalid fish (Fig. 4). The phylogenetic analyses showed that *N. shonanmaruae* is a sister species of *Narcetes erimelas* known from the Pacific, Eastern Central Atlantic, and Indian Ocean at depths of between 1300 and 2740 m^10,13^ (Fig. 4). The genetic distances between the *COI* gene sequences of *N. shonanmaruae* and *Narcetes erimelas* are 0.017–0.018, which are greater than the intraspecific values in *N. shonanmaruae* (0.000–0.002). The bootstrap value in the ML analysis (94%) indicated the monophyly of this clade. The monophyly of the order Alepocephaliformes was supported by a bootstrap value of 100%. The family Alepocephalidae was not monophyletic due to the phylogenetic position of *Bathylaco nigricans* that fell into a single clade with four species from the family Platytroctidae, and the bootstrap value did not strongly support monophyly (Fig. 4).

### Stomach content analysis

Stomach contents were analysed from two individuals of *N. shonanmaruae* (SH8-69 and SH8-43), both of which contained chyme. In addition, the stomach of the holotype (SH8-69) contained a pair of fish otoliths (~ 5 mm in diameter). However, due to the advanced state of degradation, it was not possible to identify the species of prey fish from these otoliths.

DNA barcoding targeting prey mitochondrial *COI* genes was performed using chyme collected from the holotype. After removal of low-quality reads, chimeric sequences, and singletons from all detected reads, ~ 200,000 reads were obtained and were clustered in three different OTUs (Supplementary Table S3). OTU1 (85% appearance in the total reads) was derived from *N. shonanmaruae* despite the use of blocking PCR primers designed to reduce amplification of the host DNA. OTU2 (9% appearance in the total reads) showed highest identity (91%) with the mitochondrial *COI* gene of the genus *Bassozetus* in the family Ophidiidae. OTU3 (6% appearance in the total reads) showed highest identity (88%) with the fungal genus *Cortinarius* (Agaricomycetes; Agaricomycetidae).

### Compound-specific isotope analysis of amino acids

**Figure 5:**
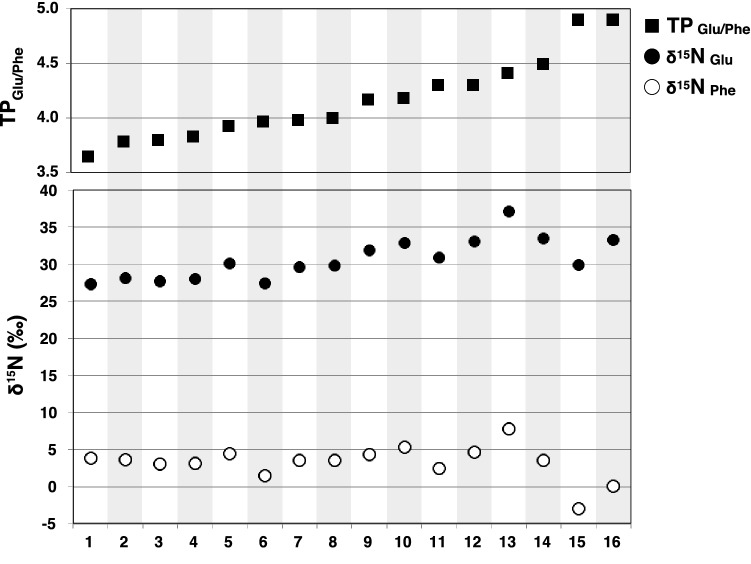
Nitrogen isotopic compositions and trophic positions of deep-sea fish collected in Suruga Bay. Solid circles: nitrogen isotopic compositions of glutamic acid, open circles: nitrogen isotopic compositions of phenylalanine, solid squares: trophic positions (TP) estimated using values of the nitrogen isotopic compositions of glutamic acid and phenylalanine (TP_Glu/Phe_). Values of organisms were based on the primary production of phytoplankton. TP_Glu/Phe_ was calculated using Eq. (1) with a *β* value of − 3.4‰. The numbers shown beneath the graph are the sample numbers indicated in Table 3.

The nitrogen isotopic composition of amino acids and the trophic positions (TPs) estimated from the values of glutamic acid and phenylalanine are shown in Table 3 and Fig. 5. The estimated TPs of the two examined *N. shonanmaruae* individuals were both 4.9, which was the highest among the predatory fishes examined in this study. The nitrogen isotopic compositions of phenylalanine from *N. shonanmaruae* (#15 and #16) (– 3.0‰ and 0.1‰, respectively) were slightly lower than those of the other fish species examined (#1–14) (+ 3.9‰, SD = 1.5, *n* = 14) (Fig. 5).

**Table 3:** The trophic position of fishes examined in this study.

No	Species	Location	Habitat	Total Length (cm)	Weight (kg)	*δ*^15^N		TP_Glu/Phe_
						Glu	Phe	
1	*Chlamydoselachus anguineus*	Suruga Bay	Bathydemersal	NA	NA	27.3	3.8	3.7
2	*Chlamydoselachus anguineus*	Suruga Bay	Bathydemersal	156.0	NA	28.1	3.6	3.8
3	*Odontaspis ferox*	Suruga Bay	Benthopelagic	162.4	NA	27.7	3.0	3.8
4	*Odontaspis ferox*	Suruga Bay	Benthopelagic	124.3	9.8	28.0	3.1	3.8
5	*Somniosus pacificus*	Suruga Bay	Benthopelagic	178.6	48.4	30.0	4.4	3.9
6	*Mitsukurina owstoni*	Suruga Bay	Bathydemersal	223.6	35.9	27.4	1.5	4.0
7	*Heptranchias perlo*	Suruga Bay	Bathydemersal	115.5	NA	29.6	3.5	4.0
8	*Mitsukurina owstoni*	Suruga Bay	Bathydemersal	212.0	23.1	29.8	3.6	4.0
9	*Heptranchias perlo*	Suruga Bay	Bathydemersal	NA	NA	31.9	4.4	4.2
10	*Hexanchus griseus*	Suruga Bay	Bathydemersal	271.6	119.1	32.9	5.3	4.2
11	*Centroscymnus owstonii*	Suruga Bay	Bathydemersal	78.6	2.6	30.9	2.4	4.3
12	*Hexanchus griseus*	Suruga Bay	Bathydemersal	NA	NA	33.1	4.6	4.3
13	*Pseudotriakis microdon*	Suruga Bay	Bathydemersal	208.4	43.0	37.1	7.8	4.4
14	*Centroscymnus owstonii*	Suruga Bay	Bathydemersal	112.2	NA	33.5	3.5	4.5
15	*Narcetes shonanmaruae* SH8-43	Suruga Bay	Benthopelagic	122.0	14.8	29.9	–3.0	4.9
16	*Narcetes shonanmaruae* SH8-69	Suruga Bay	Benthopelagic	138.0	22.6	33.3	0.1	4.9

Positions were calculated using the nitrogen isotopic compositions of glutamic acid and phenylalanine using the following equation: TP_Glu/Phe_ = (δ^15^N_Glu_ – δ^15^N_Phe_ – 3.4)/7.6 + 1.

### In situ observation of *Narcetes shonanmaruae* using a baited camera

Four baited camera casts were conducted at depths greater than 2000 m in Suruga Bay (~ 16 h of video footage in total), and a single individual of *N. shonanmaruae* was observed in the bay mouth on 26th November 2016 during cast no. AT2-04 at a depth of 2572 m (Figs. 1, 2g, Supplementary Table S4, Supplementary Video S1). A 12-s sequence of high-definition video footage showed that this *N. shonanmaruae* individual swam into the video frame from the right side and then changed direction towards a position out of view of the camera lens (Supplementary Video S1). The fish vigorously beat its tail fin when veering. Its total length estimated from the video image was more than 100 cm. Individuals of the pudgy cusk-eel *Spectrunculus grandis* and lithodid crab *Paralomis* sp. were also observed in the video recordings, as shown in Supplementary Video S1.

## Discussion

In this paper, we describe a novel slickhead fish species, which was collected from deep-water sites within Suruga Bay, Japan. To the best of our knowledge, this species is the largest among species in the family Alepocephalidae, and its trophic position is one of the highest among marine organisms reported globally to date^13,16,17^.

Maximum standard lengths (SL) of 77 species from the family are presented in FishBase and a previous study^10,13,16^ (summarized in Supplementary Table S5). A frequency distribution of SL in Alepocephalidae clearly shows that *N. shonanmaruae* and two other slickheads (*Alepocephalus bairdii* and *A. agassizii*) are much larger compared to other species in the family, exceeding 100 cm in SL (Supplementary Fig. S17). The length range with highest frequency in SL is 20–40 cm, and the average maximum SL is 35.3 cm (*n* = 78, SD = 21.5, including the present species), which demonstrate the large size of *N. shonanmaruae*.

Deep-sea gigantism, which was first described for a plaice species *Pleuronectes platessa* showing a size increase with depth^18^, has previously been reported in various marine taxa, e.g. the giant squid *Architeuthis dux*, the giant deep-sea isopod *Bathynomus giganteus*, the Japanese spider crab *Macrocheira kaempferi,* the bluntnose sixgill shark *Hexanchus griseus*, and the oarfish *Regalecus glesne*^19^. Although there is currently no generally accepted explanation for the deep-sea gigantism observed in fishes, it has been proposed that, in crustaceans, this phenomenon is a consequence of larger cell size that develops in response to lower temperatures^20^, as has also been proposed for other groups^21^. In crustaceans, deep-sea gigantism may also in part reflect decreases in temperature leading to a longer lifespan and thus larger sizes for species with indeterminate growth^20^. Among fish taxa, scavenging species, identified as those that regularly appear at baited cameras, increase in size with depth, whereas non-scavenging species decrease in size over the same depth range^22,23^. The gigantism of deep-sea fish could be interpreted in terms of a response to differences in the characteristics of food supply by the two groups^22^. Scavenging species depend on large, randomly distributed packages of carrion falling from the shallower waters, and a larger body size permits greater swimming abilities and endurance, thereby allowing the fish to move efficiently between occasional feeding events in the deep sea^23^. Trophic level and body size are also believed to be positively correlated across all the species examined, and morphological constraints associated with gape limitation may play a prominent role in determining body size^24^. Deep-sea alepocephaliform fishes are highly diverse, particularly within the family Alepocephalidae (slickheads), which consists of more than 90 species recorded globally^25–27^. Slickheads have been suggested to be important consumers of gelatinous zooplankton^28–30^, and generally show lower trophic positions than that of other fishes living in the same environments^31,32^. Thus, the feeding ecology and the trophic level of *N. shonanmaruae* would appear to be unique in this family. The scavenging ability (as implied by capture using a longline) and broad gape of *N. shonanmaruae* (Supplementary Fig. S16f) might be correlated with its colossal body size and relatively high trophic position.

The estimated trophic positions (TP = 4.9) of the two individuals for which stomach contents were analysed were the highest among the predators examined in the present study and are relatively high compared with the values obtained in previous studies^17,33–36^. The bluntnose sixgill shark *H. griseus* is known as an apex predator/scavenger in the deep sea^37–39^, whereas the false catshark *Pseudotriakis microdon* is a large predatory shark that reaches 3 m in length and feeds on a wide variety of prey, including teleost and chondrichthyan fishes, cephalopods, and marine mammals^40^. The trophic positions determined for *H. griseus* (4.2 and 4.3) and *P. microdon* (4.3) in the present study are all lower than that of *N. shonanmaruae*. Since trophic positions estimated from stable isotope analysis provide long-term dietary information^41,42^, these results indicate that *N. shonanmaruae* commonly feeds on high-trophic prey (TP = ca. 4). Nielsen et al.^17^ compiled amino acid stable nitrogen isotope ratios from 359 marine species covering four trophic levels (primary producer, herbivore, omnivore, and carnivore) from the literature. We calculated all the TPs in this previous study using Eq. (1) shown in the Methods section with *β* values of – 3.4‰. The highest TP among all taxa listed was observed in an unidentified dogfish shark *Squalus* sp. (TP = 4.8), and the highest in a teleost was observed in a moray eel *Gymnothorax kidako* (TP = 4.7). Both species are aggressive predators, and the TP values are comparable to that of *N. shonanmaruae*. Although further information is needed on the trophic positions of other species from the deepest waters of Suruga Bay in order to clarify the relative position of *N. shonanmaruae* in the bathyal ecosystem, there is little doubt regarding the relatively high trophic position of this slickhead in marine environments.

The nitrogen isotopic compositions of phenylalanine from *N. shonanmaruae* were slightly lower than those of the other fish species examined (Fig. 5). Such differences are within intra/inter-species variations collected from same regions. Intra-species variations of phenylalanine δ^15^N values were reported from a largescale blackfish *Girella punctata* collected sympatrically in Sagami Bay, Japan (the values ranging from 3.7 to 8.7), but the TPs were nearly consistent (TP ranging from 2.7 to 3.1) because the nitrogen isotopic compositions of glutamic acid fluctuated according to those of phenylalanine^36^. The phenylalanine δ^15^N values were also variable between planktonic/pelagic taxa collected at a same site 100 km north of the island of Oahu in the North Pacific Subtropical Gyre^43^, ranging from − 4.8 to 7.5, which is comparable to the values found in the results of the present study (Fig. 5).

Both morphological analyses and DNA barcoding of stomach contents showed *N. shonanmaruae* to be piscivorous in its feeding habits. In this regard, although a pair of fish otoliths was found in the stomach of the holotype, prey species identification was not possible due to the severe degradation of these otoliths. Even though the stomachs of both analysed individuals were almost empty, small amounts of chyme were detected. Such a paucity of stomach contents is common among deep-sea fish that tend to regurgitate their stomach contents upon being landed^44^. DNA meta-barcoding of the stomach chyme also supported a piscivorous habit. A sequence similarity search for sequences amplified from the chyme indicated the prey item to be an ophidiid fish (Family Ophidiidae), which comprise a dominant group of deep-sea demersal fishes containing 218 known species^45,46^. This result is consistent with a predatory lifestyle. *N. shonanmaruae* possesses large, widely spaced gill rakers (Fig. 2f, Supplementary Fig. S16c). In general, gill rakers play the role of entrapment structures to retain planktonic prey from a volume of engulfed water and are thus common in planktivorous filter feeding fish^47,48^. Increasing the number of gill rakers enhances crossflow filtering and the closely spaced gill rakers also limit the escape possibilities of small prey. However, a dense gill raker apparatus is more likely to become clogged by sediments than sparser gill rakers, and foraging in the muddy bottom of the profundal zone most likely requires other gill raker adaptations. Accordingly, a high number of long gill rakers is common in planktivorous fish species, whereas benthic species usually display a lower number of shorter gill rakers^49–52^. The rakers of *N. shonanmaruae* seem to be too sparse to support filter feeding, which is inconsistent with a planktivorous feeding habit.

Cannibalism of this slickhead might be possible, because the most dominant OTU from DNA meta-barcoding of the stomach chyme was the sequences of *N. shonanmaruae* even though a blocking primer against the *N. shonanmaruae* sequence was applied. However, several previous studies showed amplification of host sequences from stomach contents in spite of using specific blocking primers against the host^53,54^. In addition, there is no record of smaller individuals of this slickhead at any depths in Suruga Bay (Y. F., unpublished data), which implies difficulty in smaller *N. shonanmaruae* individuals being the staple diet for adults. Somewhat incongruously, OTU3 showed high homology with several species of Agaricomycetes (Fungi) and Oomycetes (Stramenopiles). As these are not considered to form part of the *N. shonanmaruae* diet, it is assumed that they could be experimental contaminants of terrestrial origin^55,56^. Alternatively, they might be unknown parasitic organisms present in the digestive apparatus of slickheads or environmental DNA included in the stomach sample.

Our video observations revealed that *N. shonanmaruae* is an active swimmer (Supplementary Video S1). In the same video sequence, we observed the most dominant fishes living at the depth (*Spectrunculus grandis*, *Simenchelys parasitica*, and *Coryphaenoides acrolepis*), which are taeniform or anguilliform species that do not move rapidly^57,58^. In contrast, the newly discovered slickhead—being fusiform and possessing a narrowed but robust caudal peduncle with a relatively large emarginate caudal fin—was observed veering vigorously with a single tail stroke and rapidly disappearing from the video frame. Such a body plan and swimming behaviour are consistent with the stomach contents (i.e. fish) and a high trophic position, all indicating that this slickhead is probably a top predator in the deepest part of Suruga Bay.

Each year, many new species of fish are discovered, most of which are relatively small^13,59^. In contrast, discoveries of large bony fishes from marine environments have been relatively rare in recent years. Since the 1800s, Suruga Bay has been one of the most studied bays in Japan^4–6^, and has yielded many marine organisms described as new species^60–62^. Deep-sea fishing, including that using longlines and bottom trawls, continues to be practiced in Suruga Bay, and therefore the deep-sea fauna inhabiting this bay is relatively well documented^7–9^. The discovery of a colossal slickhead from this bay was thus completely unexpected. In the present study, we conducted two longline searches at depths greater than 2171 m during two separate cruises and collected two individuals from each line. This high encounter probability might be due to a combination of the research method used and depth searched. Longline fishing is generally conducted at shallower depths (shallower than 1000 m in Suruga Bay) because of the difficulty of line control and limited profitable catches. Scientific trawls and dredges have sometimes been conducted at greater depths, although it is difficult to collect large, fast swimmers. In this regard, obvious differences in size and species selectivity of longlines and trawl nets have been reported ^63^, and it is highly likely that further longline-based surveys in deep waters will reveal the actual diversity of deep-sea predators, not only in Suruga Bay but also in many marine environments worldwide.

Top predators play an important role in the maintenance of species diversity and of ecosystem functions^64–67^. The extinction and reintroduction of gray wolves in Yellowstone National Park is an example of the drastic change in a population of top predators affecting the entire ecosystem^68^. Similar instances are too frequent to enumerate not only in terrestrial but also in marine ecosystems^65,69,70^. “Fishing down the food web”^71^ occurs even in the deep sea that is the largest habitat for life on the Earth. Trophic positions of catches from deep sea are quite high (from 3.5 to 4.5 predominantly, estimated using FishBase), and some fish resources have already been depleted due to over fishing^72^. Oceanic warming, acidification, and deoxygenation are occurring even in the deep sea^1^, which is assumed to initially affect large, predatory consumers. If the deep-sea ecosystems are severely damaged through the trophic cascade like that in Yellowstone, its influence on the global environment is immeasurable. As an initial step, there is an urgent need to conduct a broad and precise census of predators in the deep oceans to facilitate protection of natural resources of the planet and human livelihoods.

## Methods

### Bottom longline

In 2016, two research cruises were conducted in January–February (SH16-01) and in November–December (SH16-02) in Suruga Bay using the training vessel *Shonan maru* belonging to the Kanagawa Prefectural Marine Science High School. Two research longlines, SH8 and SH12, were deployed on the bottom of the Suruga Trough at the mouth of the bay on 3rd and 4th February and 22nd and 23rd November 2016, respectively (Fig. 1). The coordinates and depths of the surveyed sites are shown in Supplementary Table S4. Both longlines were left overnight on the deep-sea floor, and then retrieved onboard.

The longlines were composed of a 4-km main line (5 mm in diameter, polyester), 400 hooks with 5-m branch lines through the main line, two 10-kg lead sinkers, and a radio buoy at each end of the main line. A half or one-third portion of mackerel was impaled on each hook as bait. Three miniature salinity, temperature, and depth loggers (DST-CTD; Star-Oddi, Garðabær, Iceland) were attached to the start, mid, and end points of the main lines. This study was conducted in accordance with the Guidelines for Proper Conduct of Animal Experiments published by the Science Council of Japan, and the guidelines for fish experiments published by the Nature Conservation Committee of the Ichthyological Society of Japan. All the field experiments were approved by the Research Safety Committee of the Japan Agency for Marine-Earth Science and Technology.

### Morphological observations and X-ray tomography scanning

The total body lengths and weights of the four alepocephaliform fish caught using longlines were measured. All the individuals were immediately frozen onboard. After completion of the cruises, additional observations and detailed measurements of individuals were performed in the laboratory. Morphological characters of this alepocephaliform fish were compared to those in previous studies^10,11,73–77^. X-ray CT scanning of two individuals (holotype and paratype 1) was conducted for observations of osteological characteristics using a Discovery 750 HD CT scanner (GE Healthcare, Waukesha, WI, USA) under the following conditions: tube voltage, 120 kV; tube current, Auto mA; section thickness, 0.625 mm; rotation time, 1.0 s; and pitch, 0.984:1. Taxonomic keys were referred from previous studies^74^.

### Microfocus X-ray CT morphological analysis of otoliths

Morphological analyses of otoliths from two individuals were conducted using a microfocus X-ray CT scanner (ScanXmate–D160TSS105; Comscantecno Co. Ltd., Kanagawa, Japan). This system applies X-rays to a sampling stage, which rotates 360 degrees, with a high-resolution setting (X-ray focus spot diameter of 0.8 µm, X-ray tube voltage of 45 kV, detector array size of 1024 × 1024, 1200 projections/360º, two-times averaging, sequential imaging, 2.0 s/projection). Spatial resolution of scanning was changed from 7 to 15 µm, depending on the size of otoliths. Reconstruction of three-dimensional (3D) tomographic images was performed with ConeCTexpress^(R)^ software (Comscantecno Co. Ltd., Kanagawa, Japan) using the convolution back-projection method. In order to avoid artefacts associated with 3D image reconstruction, noise-cancelling and ring-artefact filters were applied. Calculations of lengths, volumes, and other morphological properties were performed using Molcer Plus imaging software (White Rabbit Corp. Inc., Tokyo, Japan).

### DNA sequencing

Total DNA was extracted from the muscle tissue of *N. shonanmaruae* specimens using a DNeasy Tissue and Blood Kit (QIAGEN Japan, Tokyo, Japan). Tissue samples of four other slickhead species were provided by Academia Sinica, Taiwan (three *Conocara* species), and Tokai University, Japan (*Talismania antillarum*), and used for DNA extraction. The sample IDs of these species are shown in Supplementary Table S1. Extracted DNA was used as a template for PCR amplification to amplify the mitogenome. Two universal primer sets for the mitochondrial *16S rRNA* gene^78^ and the *COI* gene^79^ were used for PCR (Supplementary Table S6). In addition, three degenerate primer sets were designed for ATPase F0 subunit 6 (*ATP6*), NADH dehydrogenase subunit 4 (*ND4*), and cytochrome *b* (*cytb*) genes (Supplementary Table S6), based on the sequences from several alepocephalid species deposited in nucleotide sequence databases. After PCR amplification and partial sequencing of the genes, additional primers specific to *N. shonanmaruae* were designed to amplify five long fragments from the mitogenome (Supplementary Table S6). Specific primers used for sequencing of the mitogenomes of the four additional slickheads were designed in the same manner. PCR was preformed using an Ex Taq Kit (TaKaRa, Kyoto, Japan). All the PCR products were purified using a Wizard SV gel and PCR purification Kit (Promega KK, Tokyo, Japan). The purified fragments were used for sequencing reactions using a Big Dye Terminator v3.1 Cycle Sequencing Kit (Applied Biosystems, Foster City, CA, USA). Sequencing was performed using an ABI PRISM 3130xl Genetic Analyzer (Applied Biosystems Japan Ltd., Tokyo, Japan). After sequencing, assembling and ambiguity editing of each sequence were conducted using CodonCode Aligner software (CodonCode Corporation, Dedham, MA, USA) to reconstruct whole mitogenome sequences.

### Phylogenetic analysis

Seventy-five OTUs (six obtained in the present study and the remainder from nucleotide sequence databases) were used for phylogenetic analysis, among which those of two Osteoglossiformes species (*Arapaima gigas* and *Osteoglossum bicirrhosum*) were used as an operational outgroup (Supplementary Table S1). All the 13 protein-coding genes (*ND1*, *ND2*, *COI*, *COII*, *ATP8*, *ATP6*, *COIII*, *ND3*, *ND4L*, *ND4*, *ND5*, *ND6*, and *cytb*) in the mitogenome were used for phylogenetic analysis. The predicted amino acid sequences of the 13 protein-coding genes were independently aligned using MAFFT v7.164b^80^ with auto parameters, followed by automatic editing of the resulting alignments using the GBLOCKS program^81,82^. An appropriate evolutionary model for each gene alignment was then selected for maximum likelihood (ML) analysis using Aminosan software^83^, based on Akaike Information Criteria (Supplementary Table S2). Amino acid sequence alignments from each gene were concatenated (3790 amino acids from 75 OTUs in total). ML analysis using the concatenated alignment of fish mitochondrial genes was performed using RAxML version 8.2.10^84^, with appropriate evolutionary models selected. ML bootstrap probability analysis was performed using the same software with 100 resamplings.

### Dietary analyses

The abdominal cavities of two *N. shonanmaruae* individuals (SH8-69 and SH8-43) were dissected prior to formalin fixation. Each stomach was excised, and the entire stomach contents were retrieved. The contents were visually examined, and prey items were selected from the digested materials. The chyme from one individual was used for DNA meta-barcoding analysis targeting residual prey DNA. DNA extraction, library preparation, and high-throughput sequencing were performed by Bioengineering Lab. Co., Ltd. (Kanagawa, Japan) as follows. Approximately 20 g of stomach chyme was freeze-dried and completely homogenized. DNA was isolated from the homogenized material using an ISOSPIN Blood & Plasmid Kit (Nippon Gene, Tokyo, Japan). Short fragments of *COI* genes were amplified using modified metazoan universal primer sets^85^, incorporating forward and reverse adapter sequences, i.e. ACACTCTTTCCCTACACGACGCTCTTCCGATCT-GGWACWGGWTGAACWGTWTAYCCYCC and GTGACTGGAGTTCAGACGTGTGCTCTTCCGATCT-TAHACTTCNGGGTGKCCRAARAATCA. In this procedure, a set of blocking primers was used to prevent amplification of the *COI* gene from the predatory (host) fish, as described previously^86^. Using the PCR products, paired-end sequence libraries were constructed according to the tailed PCR method, and these libraries were sequenced using Illumina MiSeq (Illumina, San Diego, CA, USA) under 2 × 250 bp conditions. After removing low-quality reads, OTUs were constructed. A homology search of each OTU was subsequently performed to identify prey animals from the stomach.

### Nitrogen isotope analysis of individual amino acids

The extraction and derivatization of amino acids and nitrogen isotope analyses of specific amino acids were conducted according to the methods described in previous studies^35,87^. Each sample was hydrolysed with 12 M HCl at 110 °C for 12 h. The hydrolysate was filtered to remove any precipitate using Pall Nanosep MF Centrifugal Devices containing a GHP Membrane (Pall, Port Washington, NY, USA), and then washed with *n*-hexane/dichloromethane (3:2, v/v) to remove hydrophobic constituents. The derivatization of amino acids was performed sequentially using thionyl chloride/2-propanol (1:4, v/v) at 110 °C for 2 h and pivaloyl chloride/dichloromethane (1:4, v/v) at 110 °C for 2 h. The Pv/iPr derivatives of the amino acids were extracted with *n*-hexane/dichloromethane (3:2, v/v). The nitrogen isotopic composition of the individual amino acids was determined by GC/C/IRMS using an Agilent Technologies 6890 N gas chromatograph (Agilent Technologies, Santa Clara, CA, USA) coupled to a Thermo Fisher Scientific Delta^plus^XP mass spectrometer (Thermo Finnigan, Bremen, Germany) with a GC-C/TC III interface^35,87^. The isotopic composition was reported as the *δ*^15^N notation relative to atmospheric N_2_ (AIR). The analytical error was better than ± 0.5‰. The TP of samples (TP_Glu/Phe_) was calculated from the *δ*^15^N values of glutamic acid (*δ*^15^N_Glu_) and phenylalanine (*δ*^15^N_Phe_) using Eq. (1) with *β* values of – 3.4‰.

1 $${\text{TP}}_{{{\text{Glu}}/{\text{Phe}}}} = \, \left[ {\left( {\delta^{{{15}}} {\text{N}}_{{{\text{Glu}}}} {-}\delta^{{{15}}} {\text{N}}_{{{\text{Phe}}}} + \beta } \right)/{7}.{6}} \right] \, + { 1}$$

### Baited camera observations

Baited camera observations were conducted on 26th November 2016 at depths greater than 2000 m in the mouth of Suruga Bay (Fig. 1, Supplementary Table S4). The camera system consisted of a high-definition video camera (KCN-EV7520SDI; Totsu Sangyo, Tokyo, Japan), an HD-SDI recorder (HDS-1601; Totsu Sangyo), an LED light array composed of 20 red LEDs (OSR5XNE3C1E; OptoSupply, Hong Kong) fixed with an epoxy resin^88^, an acoustic releaser (STD-301; Kaiyo Denshi, Saitama, Japan), a sinker-releasing unit (PJ-0001; Pearl Giken, Chiba, Japan), a DST-CTD logger (Star-Oddi), a current profiler (Zpulse Doppler current sensor 4930; Aanderaa, Bergen, Norway), and two batteries (9200WP-L; Daiwa, Tokyo, Japan). The camera system was deployed from *Shonan maru* in free-fall mode. A 30-kg ballast was released by the acoustic releaser according to the release command from the ship. Video recording was started 30 min before landing and yielded a 5-h video footage. All the data, including videos and current profiles, were recovered when the system was retrieved onboard.

### Comparative material (X-rays available for all specimens, upon request)

- *Narcetes erimelas*: ZMUB uncat. (Mar-eco 3948), 237.2 mm SL, ZMUB uncat. (Mar-eco 3691), 271.0 mm SL.
- *Narcetes erimelas*: NSMT-P63883, 218.0 mm SL.
- *Narcetes erimelas* (originally described as *Bathytroctes alveatus*): MCZ 28477, 152.2 mm SL (measured from X-ray image).
- *Bathytroctes michaelsarsi*: ZMUB 16003 (Mar-eco 7297), 401.5 mm SL.
- *Bathytroctes macrolepis*: ZMUB 15996 (Mar-eco 11860-1), 247 mm SL; ZMUB uncat. (Mar-eco 11860-2), 228.0 mm SL; ZMUB uncat. (Mar-eco 11860-3), 227.5 mm SL; ZMUB uncat. (Mar-eco 3403), 330 mm SL; ZMUB uncat. (Mar-eco 2711), 271 mm SL; ZMUB 15995 (Mar-eco 3904), 185.2 mm SL.
- *Rinoctes nasutus*: ZMUB 18501 (Mar-eco 8445-1), 188.0 mm SL; ZMUB uncat. (Mar-eco 8445-2), 153.5 mm SL.
- *Leptochilichthys agassizii*: BSKU 43180, 298.2 mm SL; ZMUB uncat. (Mar-eco 13309), 133.8 mm SL; ZMUB 18460 (Mar-eco 7325), 259.0 mm SL.
- *Bathylaco nigricans*: ZMUB 18461 (Mar-eco 4401), 254.2 mm SL.

## Supplementary Information

Supplementary Figure S1.

Supplementary Figure S2.

Supplementary Figure S3.

Supplementary Figure S4.

Supplementary Figure S5.

Supplementary Figure S6.

Supplementary Figure S7.

Supplementary Figure S8.

Supplementary Figure S9.

Supplementary Figure S10.

Supplementary Figure S11.

Supplementary Figure S12.

Supplementary Figure S13.

Supplementary Figure S14.

Supplementary Figure S15.

Supplementary Figure S16.

Supplementary Figure S17.

Supplementary Tables.

Supplementary Video S1.

## Data Availability

The datasets generated and analysed during this study are available from the corresponding author upon request.
