# Supplementary material for: Discovery of a colossal slickhead (Alepocephaliformes: Alepocephalidae): an active-swimming top predator in the deep waters of Suruga Bay, Japan
Source: Sci Rep. 2021 Jan 25;11:2490. doi: 10.1038/s41598-020-80203-6 (PMC7835233; doi:10.1038/s41598-020-80203-6)

Supplementary figure S10. *Narcetes shonanmaruae*. Computed tomographic image of pelvic fin. a: holotype (SH8-69), b: paratype 1 (SH8-43). Yellow dot: pelvic fin ray, Red dot: pair of #1 pelvic fin ray. The scale is shown in Fig. 2.

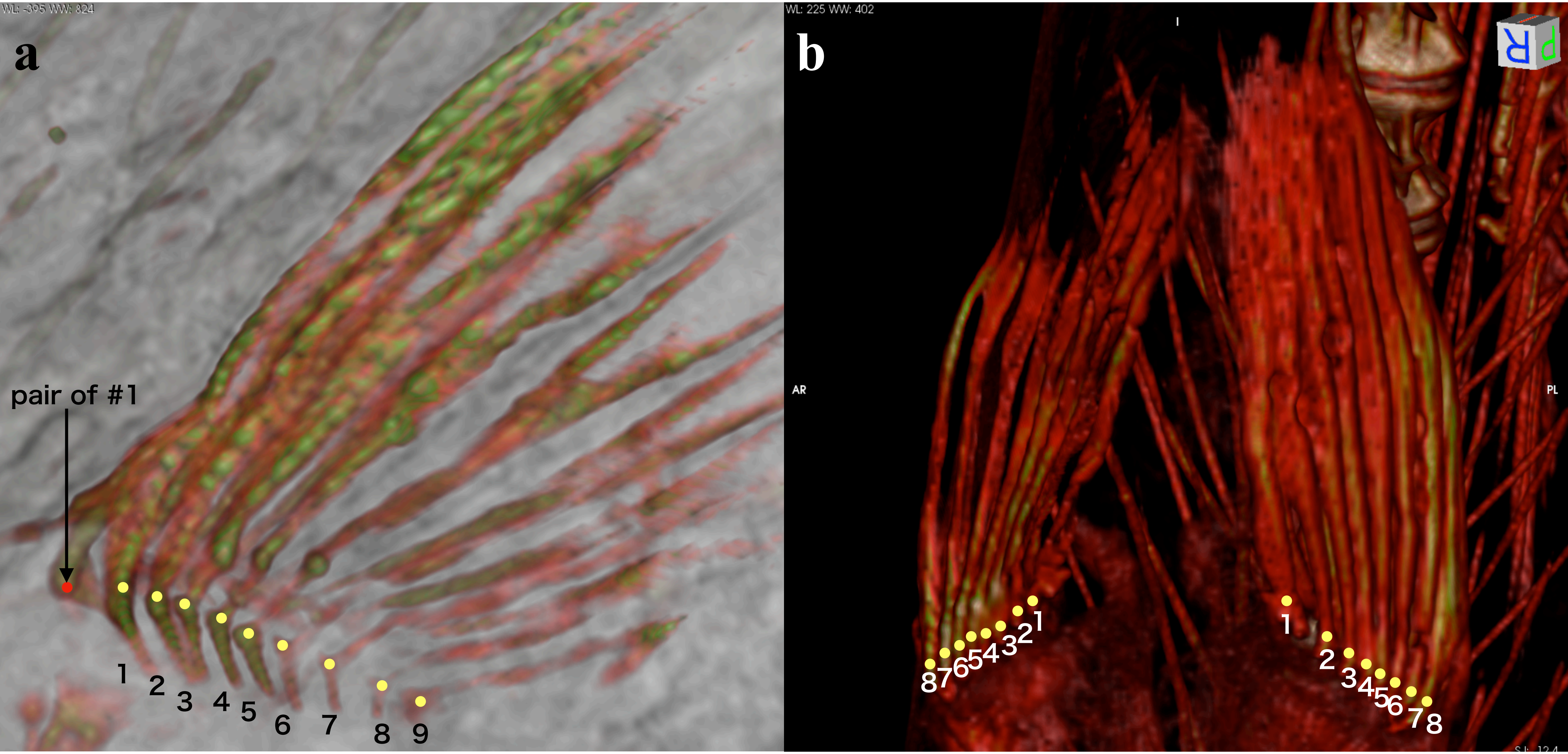

Supplement: Supplementary file 10 — Supplementary Figure S10. [file 41598_2020_80203_MOESM10_ESM.pdf]
