# Supplementary material for: Discovery of a colossal slickhead (Alepocephaliformes: Alepocephalidae): an active-swimming top predator in the deep waters of Suruga Bay, Japan
Source: Sci Rep. 2021 Jan 25;11:2490. doi: 10.1038/s41598-020-80203-6 (PMC7835233; doi:10.1038/s41598-020-80203-6)

Supplementary figure S15. *Narcetes shonanmaruae*. Three-dimensional micro-computed tomographic (CT) image of the right otolith of the paratype 1 (SH8-43). The scale is shown in Fig. 3.

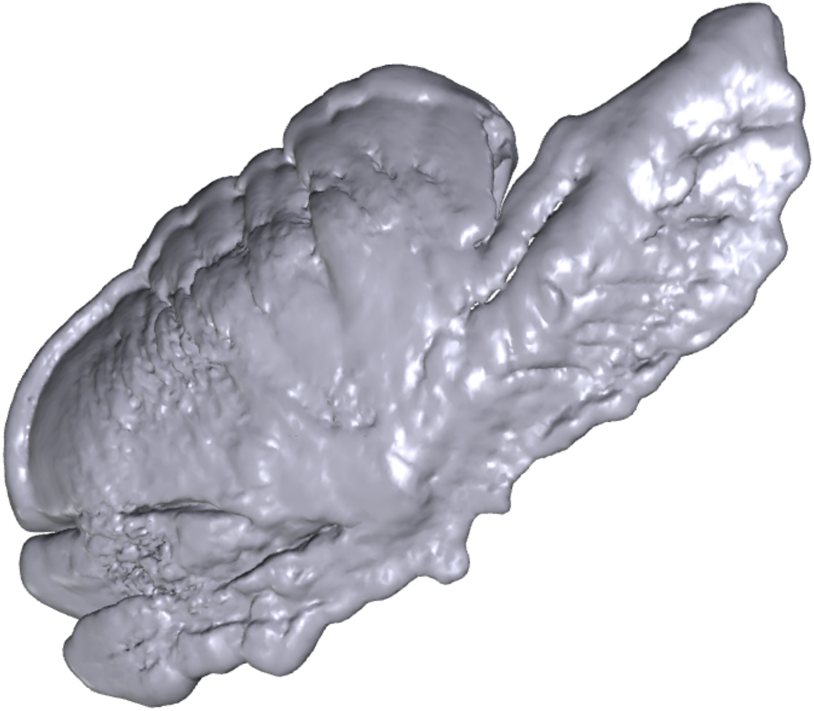

Supplement: Supplementary file 15 — Supplementary Figure S15. [file 41598_2020_80203_MOESM15_ESM.pdf]
