# Supplementary material for: Discovery of a colossal slickhead (Alepocephaliformes: Alepocephalidae): an active-swimming top predator in the deep waters of Suruga Bay, Japan
Source: Sci Rep. 2021 Jan 25;11:2490. doi: 10.1038/s41598-020-80203-6 (PMC7835233; doi:10.1038/s41598-020-80203-6)

Supplementary figure S16. *Narcetes shonanmaruae.* (a) Lateral line scale from below the mid-dorsal base, anterior on the left, (b) Scale from body below the mid-dorsal base, anterior on the left, (c) Gill rakers and gill filaments, anterior on the upper left, (d) Pyloric caeca, (e) Ovary, (f) Extensive dentition on upper and lower jaws.

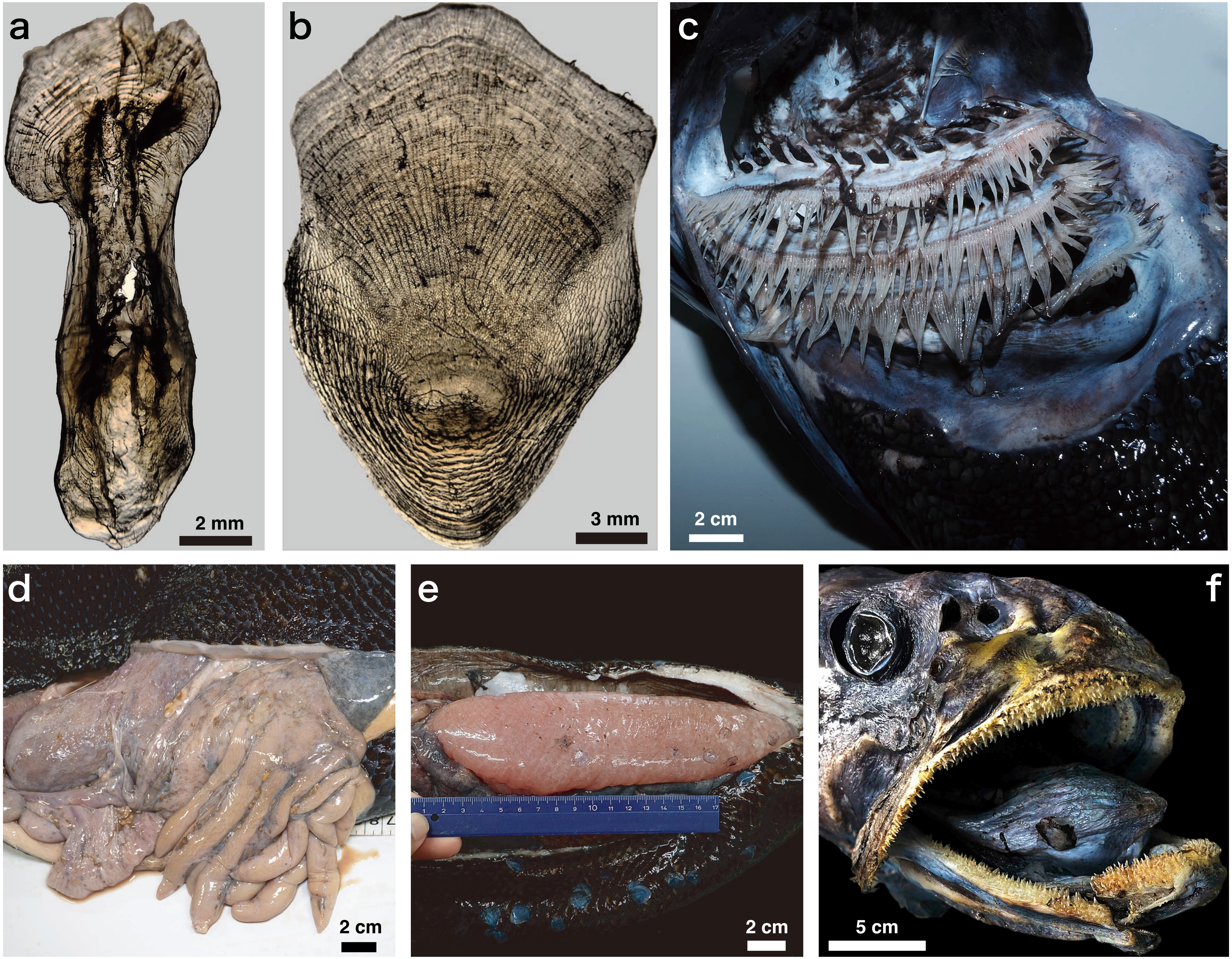

Supplement: Supplementary file 16 — Supplementary Figure S16. [file 41598_2020_80203_MOESM16_ESM.pdf]
