# Supplementary material for: Discovery of a colossal slickhead (Alepocephaliformes: Alepocephalidae): an active-swimming top predator in the deep waters of Suruga Bay, Japan
Source: Sci Rep. 2021 Jan 25;11:2490. doi: 10.1038/s41598-020-80203-6 (PMC7835233; doi:10.1038/s41598-020-80203-6)
Supplement: Supplementary file 18 — Supplementary Tables. [file 41598_2020_80203_MOESM18_ESM.pdf]

# Discovery of a colossal slickhead (Alepocephaliformes: Alepocephalidae): An active-swimming top predator in the deep waters of Suruga Bay, Japan

Yoshihiro Fujiwara[1, †, *], Masaru Kawato[1, †], Jan Yde Poulsen[2], Hitoshi Ida[3], Yoshito Chikaraishi[4, 5], Naohiko Ohkouchi[5], Kazumasa Oguri[1], Shinpei Goto[6], Genki Ozawa[1,7], Sho Tanaka[8], Masaki Miya[9], Tetsuya Sado[9], Katsunori Kimoto[1], Takashi Toyofuku[10], Shinji Tsuchida[1]

[1] Research Institute for Global Change, Japan Agency for Marine-Earth Science and Technology (JAMSTEC), Yokosuka, Kanagawa, Japan

[2] Fish Section, Australian Museum, Sydney, NSW, Australia

[3] School of Marine BioSciences, Kitasato University, Sagamihara, Kanagawa, Japan

[4] Institute of Low Temperature Science, Hokkaido University, Sapporo, Hokkaido, Japan

[5] Research Institute for Marine Resources Utilization, Japan Agency for Marine-Earth Science and Technology (JAMSTEC), Yokosuka, Kanagawa, Japan

[6] Department of Marine Electronics and Mechanical Engineering, Tokyo University of Marine Science and Technology, Koto-ku, Tokyo, Japan

[7] TechnoSuruga Laboratory Co., Ltd., Shizuoka, Shizuoka, Japan

[8] Department of Marine Biology, Tokai University, Shizuoka, Shizuoka, Japan

[9] Department of Ecology and Environmental Sciences, Natural History Museum and Institute, Chiba, Chuo-ku, Chiba, Japan

- [10] Marine Science and Technology Strategy Department, Japan Agency for Marine-Earth Science and Technology (JAMSTEC), Yokosuka, Kanagawa, Japan

†These authors contributed equally to this work.

* Corresponding author: fujiwara@jamstec.go.jp

Supplementary Table S1. Operational taxonomic units (OTUs) examined in this study for inferring phylogenetic relationships using mitogenomic sequences. *: sequenced in this study.

| OTU examined | Accession No |
|---|---|
| **Alepocephaliformes** | |
| **Alepocephalidae** | |
| *Alepocephalus agassizii* | AP009570 |
| *Alepocephalus australis* | AP009571 |
| *Alepocephalus bairdii* | AP009572 |
| *Alepocephalus bicolor* | AP009399 |
| *Alepocephalus longiceps* | AP009573 |
| *Alepocephalus productus* | AP009574 |
| *Alepocephalus tenebrosus* | AP004100 |
| *Alepocephalus umbriceps* | AP009575 |
| *Bajacalifornia megalops* | AP009578 |
| *Bathylaco nigricans* | AP009494 |
| *Bathyprion danae* | AP009400 |
| *Bathytroctes breviceps* | AP009576 |
| *Bathytroctes microlepis* | AP009401 |
| *Conocara kreffti* ASIZP P0914031* | AP018415 |
| *Conocara macropterum* | AP009580 |
| *Conocara murrayi* | AP009581 |
| *Conocara nigrum* ASIZP P0913744* | AP018413 |
| *Conocara* sp. ASIZP P0913760* | AP018414 |
| *Herwigia kreffti* | AP009582 |
| *Leptoderma lubricum* | AP009583 |
| *Leptoderma macrophthalmum* | AP011500 |
| *Leptoderma retropinna* | AP009584 |
| *Leptochilichthys agassizii* | AP009403 |
| *Narcetes erimelas* | AP009405 |
| *Narcetes shonanmaruae* SH8-69* | AP018429 |
| *Narcetes shonanmaruae* SH8-43* | AP018430 |
| *Narcetes stomias* | AP009585 |
| *Nomoctes michaelsarsi* | AP009579 |
| *Nomoctes macrolepis* | AP009577 |
| *Rinoctes nasutus* | AP009586 |
| *Talismania bifurcata* | AP009587 |
| *Talismania antillarum* Tokai S080716* | AP018416 |
| *Xenodermichthys copei* | AP009588 |
| **Platytroctidae** | |
| *Maulisia argipalla* | AP009404 |
| *Normichthys operosus* | AP009406 |
| *Playtroctes apus* | AP004107 |
| *Sagamichthys abei* | AP009495 |
| **Albuliformes** | |
| *Albula glossodonta* | AP002973 |
| **Anguilliformes** | |
| *Anguilla japonica* | KJ948424 |
| *Gymnomuraena zebra* | KP793920 |
| **Argentiniformes** | |
| *Bathylychnops exilis* | AP012953 |
| *Dolichopteryx minuscula* | AP012954 |
| **Ateleopodiformes** | |
| *Ateleopus japonicus* | AP002916 |
| *Ijimaia dofleini* | AP002917 |

| OTU examined | Accession No |
|---|---|
| **Aulopiformes** | |
| *Aulopus japonicus* | AB047821 |
| *Saurida undosquamis* | KJ511779 |
| **Characiformes** | |
| *Chalceus macrolepidotus* | AB054130 |
| **Clupeiformes** | |
| *Alosa alosa* | AP009131 |
| *Clupea harengus* | AP009133 |
| *Ilisha elongata* | AP009141 |
| **Cypriniformes** | |
| *Gobio gobio* | AB239596 |
| *Myxocyprinus asiaticus* | AB223007 |
| **Elopiformes** | |
| *Elops saurus* | AP004807 |
| *Megalops atlanticus* | AP004808 |
| **Esociformes** | |
| *Dallia pectoralis* | AP004102 |
| *Esox lucius* | AP004103 |
| **Gadiformes** | |
| *Lota lota* | KM363244 |
| **Gonorynchiformes** | |
| *Cromeria nilotica* | AP011560 |
| *Grasseichthys gabonensis* | AP007277 |
| *Kneria* sp. SL-2004 | AP007278 |
| *Parakneria cameronensis* | AP007279 |
| *Phractolaemus ansorgii* | AP007280 |
| **Gymnotiformes** | |
| *Eigenmannia* sp. CBM-ZF-10620 | AB054131 |
| **Notacanthiformes** | |
| *Aldrovandia affinis* | AP002974 |
| *Halosauropsis macrochir* | AP018130 |
| **Ophidiiformes** | |
| *Lamprogrammus niger* | AP004410 |
| **Osmeriformes** | |
| *Hypomesus olidus* | KP281293 |
| *Salanx ariakensis* | KM517200 |
| **Osteoglossiformes** | |
| *Arapaima gigas* | AP009497 |
| *Osteoglossum bicirrhosum* | AB043025 |
| **Perciformes** | |
| *Sillago sihama* | KR363150 |
| **Scorpaeniformes** | |
| *Hemilepidotus gilberti* | KX156764 |
| *Trachidermus fasciatus* | JX017305 |
| **Siluriformes** | |
| *Pangasianodon gigas* | AY762971 |
| **Stomiiformes** | |
| *Tactostoma macropus* | LC377784 |
| **Gonorynchiformes** | |
| *Chanos chanos* | AB054133 |
| *Gonorynchus greyi* | AB054134 |
| **Clupeiformes** | |
| *Clupea harengus* | AP009133 |
| *Denticeps clupeoides* | AP007276 |

Supplementary Table S2. Evolutionary models selected for each gene for conducting maximum likelihood analyses

| Gene | Selected evolutionary model |
|---|---|
| *ND1* | MTMAM |
| *ND2* | MTMAMF |
| *COI* | LG4XF |
| *COII* | MTMAMF |
| *ATP8* | MTMAM |
| *ATP6* | MTZOAF |
| *COIII* | MTZOAF |
| *ND3* | MTMAM |
| *ND4L* | MTMAM |
| *ND4* | MTMAM |
| *ND5* | MTMAMF |
| *ND6* | MTREVF |
| *cytb* | MTMAMF |

Supplementary Table S3. Homology search results of mitochondrial *COI* gene sequences derived from the stomach contents of *Narcetes shonanmaruae*

| | Number of reads (% of total reads) | Highest homology | Accession No. | Identity | E-value |
|---|---|---|---|---|---|
| OTU1 | 179129 (84.7%) | Actinopterygii, Chordata *N. shonanmaruae* (host) | - | - | - |
| OTU2 | 12906 (9.2%) | Actinopterygii, Chordata *Bassozetus compressus* | KU885679 | 91.9% | 9e-82 |
| OTU3 | 19530 (6.1%) | Agaricomycetes, Basidiomycota *Cortinarius* sp. | JN029423 | 88.5% | 5e-74 |

Supplementary Table S4. Research coordinates and depths. (S) start and (E) end locations of longlines

| Date | Research type | No. | Latitude (N) | Longitude (E) | Depth (m) |
|---|---|---|---|---|---|
| 3rd/4th Feb. 2016 | Longline | SH8 | (S) 34°39.50′<br>(E) 34°41.77′ | (S) 138°33.90′<br>(E) 138°34.01′ | (S) 2171<br>(E) 1882 |
| 22nd/23rd Nov. 2016 | Longline | SH12 | (S) 34°35.64′<br>(E) 34°33.40′ | (S) 138°34.67′<br>(E) 138°34.65′ | (S) 2532<br>(E) 2572 |
| 26th Nov. 2016 | Baited camera | AT2-04 | 34°33.74′ | 138°33.77′ | 2572 |

Supplementary Table S5. List of maximum standard length (SL) in the family Alepocephalidae

| Species | Max SL (cm) | Reference for body length | Remarks |
|---|---|---|---|
| *Narcetes shonanmaruae* | 121.0 | This study | |
| *Alepocephalus bairdii* | 117.0 | Mindel et al., 2016 | Max SL caliculated from TL |
| *Alepocephalus agassizii* | 108.0 | Mindel et al., 2016 | Max SL caliculated from TL |
| *Narcetes erimelas* | 74.7 | Sazonov (1998) | |
| *Conocara salmoneum* | 73.0 | FishBase (Dec. 2018) | |
| *Alepocephalus australis* | 69.0 | FishBase (Dec. 2018) | |
| *Alepocephalus antipodianus* | 66.2 | FishBase (Dec. 2018) | |
| *Alepocephalus umbriceps* | 63.0 | FishBase (Dec. 2018) | |
| *Bajacalifornia megalops* | 58.0 | Mindel et al., 2016 | Max SL caliculated from TL |
| *Narcetes stomias* | 57.5 | Sazonov (1998) | |
| *Alepocephalus rostratus* | 52.0 | Mindel et al., 2016 | Max SL caliculated from TL |
| *Rouleina attrita* | 52.0 | Mindel et al., 2016 | Max SL caliculated from TL |
| *Narcetes lloydi* | 50.0 | FishBase (Dec. 2018) | |
| *Talismania longifilis* | 46.3 | FishBase (Dec. 2018) | |
| *Conocara murrayi* | 45.0 | Mindel et al., 2016 | Max SL caliculated from TL |
| *Alepocephalus longirostris* | 41.0 | FishBase (Dec. 2018) | |
| *Alepocephalus productus* | 41.0 | FishBase (Dec. 2018) | |
| *Alepocephalus owstoni* | 40.0 | FishBase (Dec. 2018) | |
| *Bathytroctes macrolepis* | 40.0 | FishBase (Dec. 2018) | |
| *Herwigia kreffti* | 40.0 | FishBase (Dec. 2018) | |
| *Conocara paxtoni* | 38.7 | FishBase (Dec. 2018) | |
| *Conocara kreffti* | 38.6 | FishBase (Dec. 2018) | |
| *Bajacalifornia calcarata* | 38.0 | FishBase (Dec. 2018) | |
| *Bathyprion danae* | 38.0 | FishBase (Dec. 2018) | |
| *Conocara macropterum* | 38.0 | Mindel et al., 2016 | Max SL caliculated from TL |
| *Rouleina squamilatera* | 38.0 | FishBase (Dec. 2018) | |
| *Conocara fiolenti* | 37.2 | FishBase (Dec. 2018) | |
| *Bathytroctes michaelsarsi* | 37.0 | FishBase (Dec. 2018) | |
| *Talismania filamentosa* | 37.0 | FishBase (Dec. 2018) | |
| *Bathylaco nigricans* | 36.0 | FishBase (Dec. 2018) | |
| *Conocara werneri* | 34.0 | FishBase (Dec. 2018) | |
| *Rouleina livida* | 34.0 | FishBase (Dec. 2018) | |
| *Asquamiceps caeruleus* | 33.7 | FishBase (Dec. 2018) | |
| *Bathytroctes microlepis* | 32.3 | FishBase (Dec. 2018) | |
| *Bathytroctes oligolepis* | 32.3 | FishBase (Dec. 2018) | |
| *Rouleina maderensis* | 32.0 | FishBase (Dec. 2018) | |
| *Alepocephalus planifrons* | 31.7 | FishBase (Dec. 2018) | |
| *Bathytroctes squamosus* | 30.6 | FishBase (Dec. 2018) | |
| *Alepocephalus dentifer* | 30.5 | FishBase (Dec. 2018) | |
| *Bathytroctes breviceps* | 30.3 | FishBase (Dec. 2018) | |
| *Bathylaco nielseni* | 29.1 | FishBase (Dec. 2018) | |
| *Alepocephalus bicolor* | 29.0 | FishBase (Dec. 2018) | |
| *Talismania homoptera* | 29.0 | FishBase (Dec. 2018) | |
| *Aulastomatomorpha phospherops* | 28.0 | FishBase (Dec. 2018) | |
| *Talismania bifurcata* | 27.7 | FishBase (Dec. 2018) | |
| *Conocara microlepis* | 27.0 | FishBase (Dec. 2018) | |
| *Talismania okinawensis* | 27.0 | FishBase (Dec. 2018) | |
| *Talismania mekistonema* | 26.7 | FishBase (Dec. 2018) | |
| *Asquamiceps hjorti* | 25.3 | FishBase (Dec. 2018) | |
| *Bajacalifornia microstoma* | 24.7 | FishBase (Dec. 2018) | |

| Species | Max SL (cm) | Reference for body length | Remarks |
|---|---|---|---|
| *Bathytroctes macrognathus* | 24.2 | FishBase (Dec. 2018) | |
| *Alepocephalus triangularis* | 24.0 | FishBase (Dec. 2018) | |
| *Leptoderma macrops* | 24.0 | FishBase (Dec. 2018) | |
| *Rouleina euryops* | 23.4 | FishBase (Dec. 2018) | |
| *Rouleina guentheri* | 23.0 | FishBase (Dec. 2018) | |
| *Talismania kotlyari* | 23.0 | FishBase (Dec. 2018) | |
| *Einara macrolepis* | 22.0 | FishBase (Dec. 2018) | |
| *Rouleina watasei* | 22.0 | FishBase (Dec. 2018) | |
| *Bathytroctes inspector* | 21.8 | FishBase (Dec. 2018) | |
| *Bajacalifornia arcylepis* | 21.6 | FishBase (Dec. 2018) | |
| *Leptoderma lubricum* | 21.0 | FishBase (Dec. 2018) | |
| *Leptoderma retropinna* | 21.0 | FishBase (Dec. 2018) | |
| *Einara edentula* | 20.3 | FishBase (Dec. 2018) | |
| *Mirognathus normani* | 20.0 | FishBase (Dec. 2018) | |
| *Xenodermichthys nodulosus* | 20.0 | FishBase (Dec. 2018) | |
| *Rinoctes nasutus* | 19.0 | FishBase (Dec. 2018) | |
| *Talismania bussingi* | 18.6 | FishBase (Dec. 2018) | |
| *Bajacalifornia burragei* | 18.1 | FishBase (Dec. 2018) | |
| *Alepocephalus fundulus* | 18.0 | FishBase (Dec. 2018) | |
| *Asquamiceps velaris* | 17.4 | FishBase (Dec. 2018) | |
| *Alepocephalus longiceps* | 16.0 | FishBase (Dec. 2018) | |
| *Talismania antillarum* | 16.0 | FishBase (Dec. 2018) | |
| *Leptoderma macrophthalmum* | 15.1 | FishBase (Dec. 2018) | |
| *Bajacalifornia aequatoris* | 14.5 | FishBase (Dec. 2018) | |
| *Rouleina nuda* | 14.4 | FishBase (Dec. 2018) | |
| *Photostylus pycnopterus* | 13.0 | FishBase (Dec. 2018) | |
| *Bathytroctes pappenheimi* | 10.8 | FishBase (Dec. 2018) | |
| *Microphotolepis schmidti* | 7.2 | FishBase (Dec. 2018) | |
| *Alepocephalus tenebrosus* | 61.0 (TL) | FishBase (Dec. 2018) | |
| *Bajacalifornia erimoensis* | 36.6 (TL) | FishBase (Dec. 2018) | |
| *Xenodermichthys copei* | 31.0 (TL) | FishBase (Dec. 2018) | |
| *Alepocephalus andersoni* | | | |
| *Alepocephalus asperifrons* | | | |
| *Alepocephalus blanfordii* | | | |
| *Alepocephalus melas* | | | |
| *Asquamiceps longmani* | | | |
| *Bathylaco macrophthalmus* | | | |
| *Bathytroctes elegans* | | | |
| *Bathytroctes zugmayeri* | | | |
| *Conocara bertelseni* | | | |
| *Conocara nigrum* | | | |
| *Leptoderma affinis* | | | |
| *Microphotolepis multipunctata* | | | |
| *Rouleina danae* | | | |
| *Rouleina eucla* | | | |
| *Talismania aphos* | | | |
| *Talismania brachycephala* | | | |

Supplementary Table S6. PCR primers used for amplification and sequencing of the mitogenome of *Narcetes shonanmaruae*

| Primer | Sequence (5′–3′) | Amplification target | Orientation | Reference |
|---|---|---|---|---|
| 16SarL | CGCCTGTTTAACAAAAACAT | *16S rRNA* | Forward | Palumbi *et al*. 2002 |
| 16SbrH | CCGGTCTGAACTCAGATCACGT | *16S rRNA* | Reverse | Palumbi *et al*. 2002 |
| LCO1490 | GGTCAACAAATCATAAAGATATTGG | *COI* | Forward | Folmer *et al*. 1994 |
| HCO2198 | TAAACTTCAGGGTGACCAAAAAATCA | *COI* | Reverse | Folmer *et al*. 1994 |
| Alepo_ATP6_F1 | CARTTTATGAGCCCCACAT | *ATP6* | Forward | This study (degenerate primer) |
| Alepo_ATP6_R2 | AYAAATGCGGCRGTGGCGAT | *ATP6* | Reverse | This study (degenerate primer) |
| Alepo_ND4_F1 | TAATYGCCTACTCCTCCGT | *ND4* | Forward | This study (degenerate primer) |
| Alepo_ND4_R1 | TAGGTGYTCTCGTGTGTGGTA | *ND4* | Reverse | This study (degenerate primer) |
| Alepo_cytb_F1 | AACGGRGCYTCYTTCTTCTT | *cytb* | Forward | This study (degenerate primer) |
| Alepo_cytb_R2 | TTCRAGWRCCTTGTTTTCG | *cytb* | Reverse | This study (degenerate primer) |
| WF1 | ATGATCCGGCTGAAGCCGATCAA | *16S rRNA–COI* | Forward | This study (specific primer) |
| WR2 | GCATGTGCTGTGACGATGACATT | *16S rRNA–COI* | Reverse | This study (specific primer) |
| WF2 | ACCATACTCCTCACAGATCGTAA | *COI–ATP6* | Forward | This study (specific primer) |
| WR3 | GCCTAGCATGTTGAGAGTAATGA | *COI–ATP6* | Reverse | This study (specific primer) |
| WF3 | GACTAACTGCCAACCTTACAGCT | *ATP6–ND4* | Forward | This study (specific primer) |
| WR4 | GGTGAATCATCAGACGGCTGTTA | *ATP6–ND4* | Reverse | This study (specific primer) |
| WF4 | GCACGAGGACTCCAAATACTATT | *ND4–cytb* | Forward | This study (specific primer) |
| WR5 | GTAGAGGTAGGATCCGTAGTATA | *ND4–cytb* | Reverse | This study (specific primer) |
| WF6 | GCTGACTCATCCGAAGCATGCAT | *cytb–16S rRNA* | Forward | This study (specific primer) |
| WR6 | TGACGTGGTTGACCTTGTGTCTT | *cytb–16S rRNA* | Reverse | This study (specific primer) |
